# Network-based genetic investigation of virulence-associated phenotypes in methicillin-resistant *Staphylococcus aureus*

**DOI:** 10.1038/s41598-018-29120-3

**Published:** 2018-07-17

**Authors:** Chan Yeong Kim, Muyoung Lee, Keehoon Lee, Sang Sun Yoon, Insuk Lee

**Affiliations:** 1 Department of Biotechnology, College of Life Science and Biotechnology, Yonsei University, Seoul, 03722 Korea; 2 Department of Microbiology and Immunology, Brain Korea 21 PLUS Project for Medical Science, Yonsei University College of Medicine, Seoul, 03722 Korea

## Abstract

*Staphylococcus aureus* is a gram-positive bacterium that causes a wide range of infections. Recently, the spread of methicillin-resistant *S*. *aureus* (MRSA) strains has seriously reduced antibiotic treatment options. Anti-virulence strategies, the objective of which is to target the virulence instead of the viability of the pathogen, have become widely accepted as a means of avoiding the emergence of new antibiotic-resistant strains. To increase the number of anti-virulence therapeutic options, it is necessary to identify as many novel virulence-associated genes as possible in MRSA. Co-functional networks have proved useful for mapping gene-to-phenotype associations in various organisms. Herein, we present StaphNet (www.inetbio.org/staphnet), a genome-scale co-functional network for an MRSA strain, *S*. *aureus* subsp. USA300_FPR3757. StaphNet, which was constructed by the integration of seven distinct types of genomics data within a Bayesian statistics framework, covers approximately 94% of the coding genome with a high degree of accuracy. We implemented a companion web server for network-based gene prioritization of the phenotypes of 31 different *S*. *aureus* strains. We demonstrated that StaphNet can effectively identify genes for virulence-associated phenotypes in MRSA. These results suggest that StaphNet can facilitate target discovery for the development of anti-virulence drugs to treat MRSA infection.

## Introduction

*Staphylococcus aureus* is an opportunistic human pathogen that can cause disorders ranging from minor skin infections to life-threatening invasive diseases^1–4^. In particular, its ability to develop resistance to antibiotic treatments makes this bacterium a global medical concern^5^. To counteract antibiotic-resistant *S*. *aureus*, researchers have developed new antibiotics, and physicians administer high doses of multiple antibiotics. These approaches work in the short term, but eventually lead to the emergence of new antibiotic-resistant strains.

Recently, there has been wide acceptance of anti-virulence strategies for dealing with antibiotic-resistant bacteria^6–8^. By attenuating the virulence of bacteria, this strategy impedes disease progression in the host and buys the immune system sufficient time to fight the pathogen. At the same time, this strategy reduces the selective pressure experienced by the bacteria, thereby slowing the emergence of new antibiotic-resistant strains^9^. *S*. *aureus* has a wide variety of virulence factors such as host cell-destroying toxins^10^, and the ability to form biofilms^11^. The identification of virulence factors is necessary for the development of effective anti-virulence strategies against bacterial pathogens.

The more virulence genes are identified, the more opportunities there are for the development of therapeutics. For example, according to the Virulence Factor Database (VFDB)^12^, as of August 2017, the major MRSA strain *S*. *aureus* subsp. USA300_FPR3757 has approximately 2,700 coding genes, 78 of which are responsible for virulence-associated phenotypes. This suggests that many more virulence genes remain to be discovered, and the efficient identification of such genes could facilitate the development of anti-virulence drugs. Network-based gene prioritization for disease research has increased in popularity^13^, and co-functional networks of various organisms including hosts and pathogens can be constructed using a Bayesian statistics approach^14^. We previously constructed a co-functional network for the opportunistic fungal pathogen *Cryptococcus neoformans*, and demonstrated its usefulness for identifying novel genes involved in fungal pathogenicity and drug resistance^15^.

To apply a similar network-based approach to identifying virulence genes in *S*. *aureus*, we constructed StaphNet, a genome-scale co-functional network for *S*. *aureus* subsp. aureus USA300_FPR3757, which is a predominantly community-acquired, methicillin-resistant (CA-MRSA) *S*. *aureus* strain found in the United States^16^. We mapped co-functional associations among USA300 genes from seven distinct types of genomic data, and integrated them using a Bayesian statistics framework into StaphNet, which contains 60,513 links and 2,674 genes (~94% of the coding genome). StaphNet proved to be highly accurate and network hubs tend to be essential genes or drug target genes. StaphNet effectively reconstructed various pathways involved in *S*. *aureus* virulence, and experimentally validated novel genes predicted for hemolysis and biofilm formation.

## Results

### Construction and assessment of StaphNet

**Figure 1:**
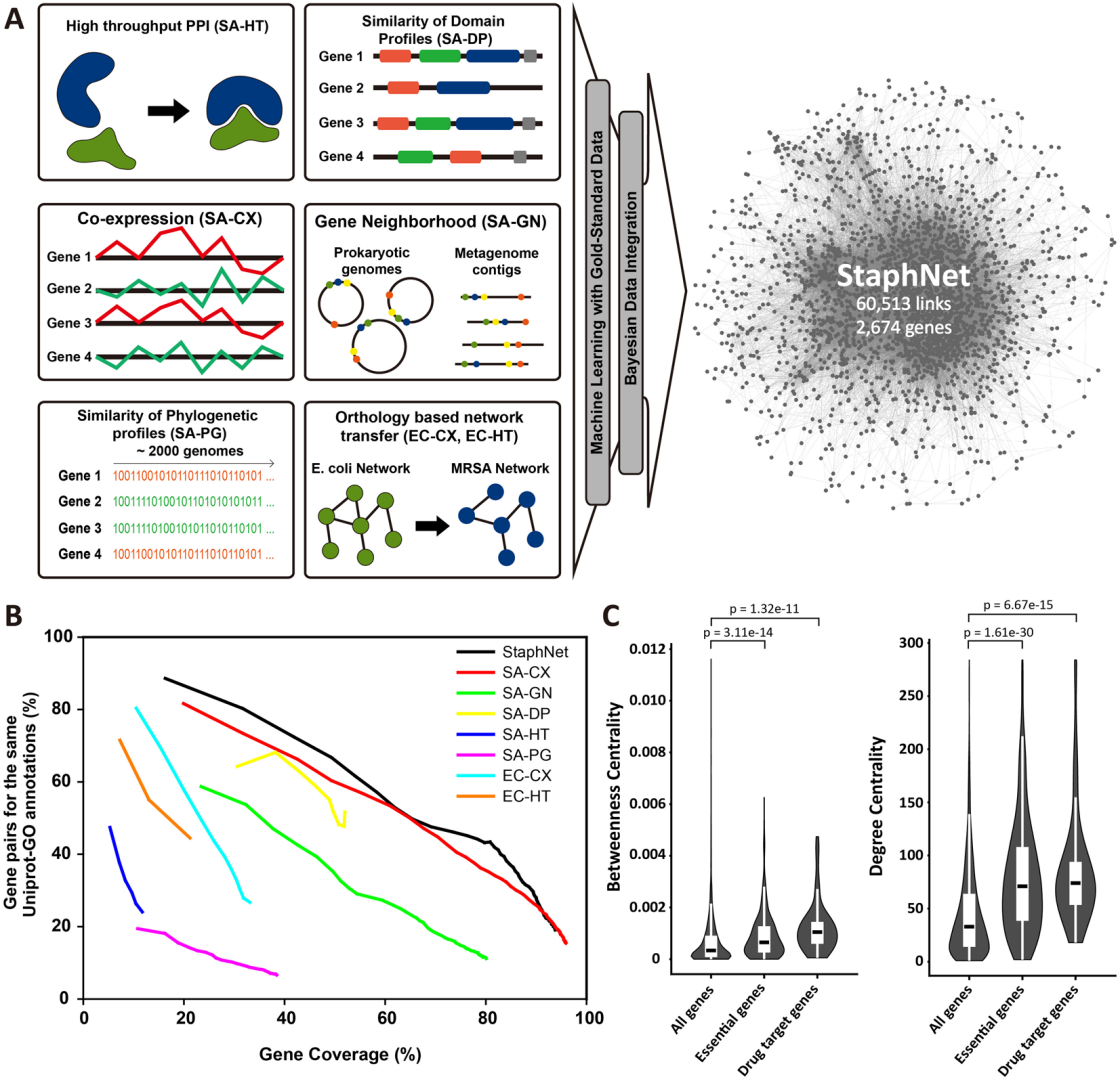
Construction and assessment of StaphNet. (**A**) Schematic overview of the process used to construct StaphNet. The functional associations were inferred from the seven distinct data types and integrated into StaphNet. (**B**) Methicillin-resistant S*taphylococcus aureus* (MRSA) and its component networks were assessed against UniProt-GO annotation. The graph represents the precision and its corresponding gene coverage for every 1,000 links. (**C**) The violin plots represent the distribution of betweenness centrality (left) and degree centrality (right) for each gene set. *P*-values were calculated using the Mann–Whitney U test.

The construction of StaphNet is described in detail in the Methods section and is summarized in Fig. 1A. Briefly, we first constructed the seven component networks from diverse genomic data sources that implicated co-functional links between genes: co-expression between *S*. *aureus* genes (SA-CX) in RNA-seq and microarray data (Table 1); high-throughput protein complex pull-down assay data in *S*. *aureus* (SA-HT)^17^, gene neighborhood (SA-GN)^18^ and similarity of phylogenetic profiles (SA-PG)^19^ across reference genomes; similarity of domain profiles (SA-DP)^20^, orthologous functional associations transferred from *Escherichia coli* gene co-expression (EC-CX); and *E*. *coli* high-throughput protein–protein interaction (EC-HT) networks^21^ (Table 2). We benchmarked and trained the seven component networks using gold-standard gene pairs compiled from the Kyoto Encyclopedia of Genes and Genomes (KEGG)^22^ and MetaCyc^23^ pathways. We then unified the intrinsic data scores using the log-likelihood score (LLS) scheme based on Bayesian statistics^24^. All these networks were then integrated into StaphNet using the weighted sum method^24^. The final network contains 60,513 co-functional links among 2,674 genes of *S*. *aureus* subsp. USA300_FPR3757, and covers 94% of the coding genome. To assess the accuracy of the network, we compiled validation gene pairs from UniProt-GO annotations^25^, and measured the proportion of gene pairs for the same GO terms. We found that the integrated network outperformed all the component networks (Fig. 1B), indicating the effectiveness of data integration in network construction.

**Table 1:** Gene Expression Omnibus (GEO) expression data used to construct StaphNet.

GSE	Platform	Description	# gene	# link
GSE26249	GPL10597	Gene expression analysis of daptomycin resistance in *Staphylococcus aureus* strains: 2818 and 2819	859	22,485
GSE10605	GPL1339	Microarray analysis of toxicogenomic effects of ortho-phenylphenol on *S*. *aureus*	882	19,510
GSE13424	GPL1339	Profiling downregulation of the mevalonate pathway in *S*. *aureus*	1,349	30,499
GSE14669	GPL1339	Transcriptional analysis of *S*. *aureus* response to ramoplanin	581	22,515
GSE15394	GPL1339	*S*. *aureus* treated with fosfomycin	945	23,492
GSE22233	GPL1339	Expression and mRNA half-life data for acid- and alkaline-shocked *S*. *aureus*	1,666	35,508
GSE3415	GPL1339	Global transcriptome analysis of *S*. *aureus* response to hydrogen peroxide	1,802	48,499
GSE39627	GPL1339	Comparison of the oxacillin stress response in vraS and vraT mutants	2,066	30,501
GSE40448	GPL1339	Microarray analysis of toxicogenomic effect of ortho-benzyl-para-chlorophenol (OBPCP) on *S*. *aureus*	1,913	58,496
GSE40450	GPL1339	Comparative analysis of the toxicogenomic effects of ortho-benzyl-para-chlorophenol (OBPCP) and para-tertiary amylphenol (PTAP) on *S*. *aureus*	1,580	45,490
GSE50675	GPL1339	Global transcriptome analysis of *S*. *aureus* biofilms in response to innate immune cells	798	22,495
GSE58938	GPL1339	Phenotype and expression profile analysis of *S*. *aureus* biofilms and planktonic cells in response to licochalcone A	904	14,504
GSE20973	GPL1339	Direct targets of CodY in *S*. *aureus*	980	17,494
GSE70040	GPL20586	Whole-transcriptome analysis of *S*. *aureus* under laboratory and infection-mimicking conditions	480	11,418
GSE25454	GPL8069	Global changes in *S*. *aureus* gene expression in human blood provide insight into mechanisms of immune evasion and virulence	1,863	44,494
GSE65827	GPL18484	SaeRS-dependent inhibition of biofilm formation in *S*. *aureus* Newman	249	9,507
GSE59851	GPL19006	Potential influence of *S*. *aureus* clonal complex 30 genotype and transcriptome on hematogenous infections	1,592	45,497
GSE68772	GPL19006	The C-terminal region of the RNA helicase CshA is required for the interaction with the degradosome and turnover of bulk RNA in the opportunistic pathogen *S*. *aureus*	1,687	18,506

**Table 2:** Summary of the seven component networks for distinct data types and the integrated StaphNet.

Network code	Description	# gene	# link
SA-CX	Co-functional network inferred by co-expression analysis of *Staphylococcus aureus* genes	2,539	24,031
SA-GN	Co-functional network inferred by gene neighborhood of *S*. *aureus* genes in prokaryotic genomes	2,031	21,803
SA-DP	Co-functional network inferred by domain profile similarity between *S*. *aureus* genes	1,448	7,026
SA-HT	Co-functional network derived from high-throughput protein–protein interaction assays for *S*. *aureus* genes	290	4,130
SA-PG	Co-functional network inferred by phylogenetic profile similarity between *S*. *aureus* genes	910	8,407
EC-CX	Co-functional network inferred by co-expression analysis of *Escherichia coli* genes	754	5,232
EC-HT	Co-functional network derived from high-throughput protein–protein interaction assays for *E*. *coli* genes	479	2,626
StaphNet	The integrated co-functional network for methicillin-resistant *S*. *aureus* (MRSA)	2,674	60,513

To test the hypothesis that gene network hubs tend to comprise essential genes^26^, we investigated the distribution of degree centrality and betweenness centrality of the genes for viability and drug targeting in StaphNet. We compiled 339 essential genes^27^ and 71 drug target genes^17^ in the USA300 strain of *S*. *aureus*. We found that drug targets and essential genes had significantly higher network centrality by both degree and betweenness (*P*-value < 1 × 10^−10^ for all comparisons, Mann–Whitney U test) (Fig. 1C). These results suggest that network centrality of genes in StaphNet can be used to predict novel genes for viability in *S*. *aureus*.

### StaphNet effectively retrieves genes for virulence-associated phenotypes

If StaphNet effectively retrieves genes for virulence-associated phenotypes, it should also be capable of identifying novel virulence genes. It is well known that *S*. *aureus* has various virulence factors. We compiled the genes associated with several virulence phenotypes such as hemolysin production, protease activity, pigment formation, and mannitol fermentation from a previous large-scale mutant screening study using *S*. *aureus* strain USA300^28^. We also compiled genes for five distinct virulence phenotypes—adherence, capsule, exoenzyme, secretion system, and toxin—from the Virulence Factor Database (VFDB)^12^. Furthermore, we compiled genes for the toxicity-increasing loci from an *S*. *aureus* genome-wide association study (GWAS)^29^.

**Figure 2:**
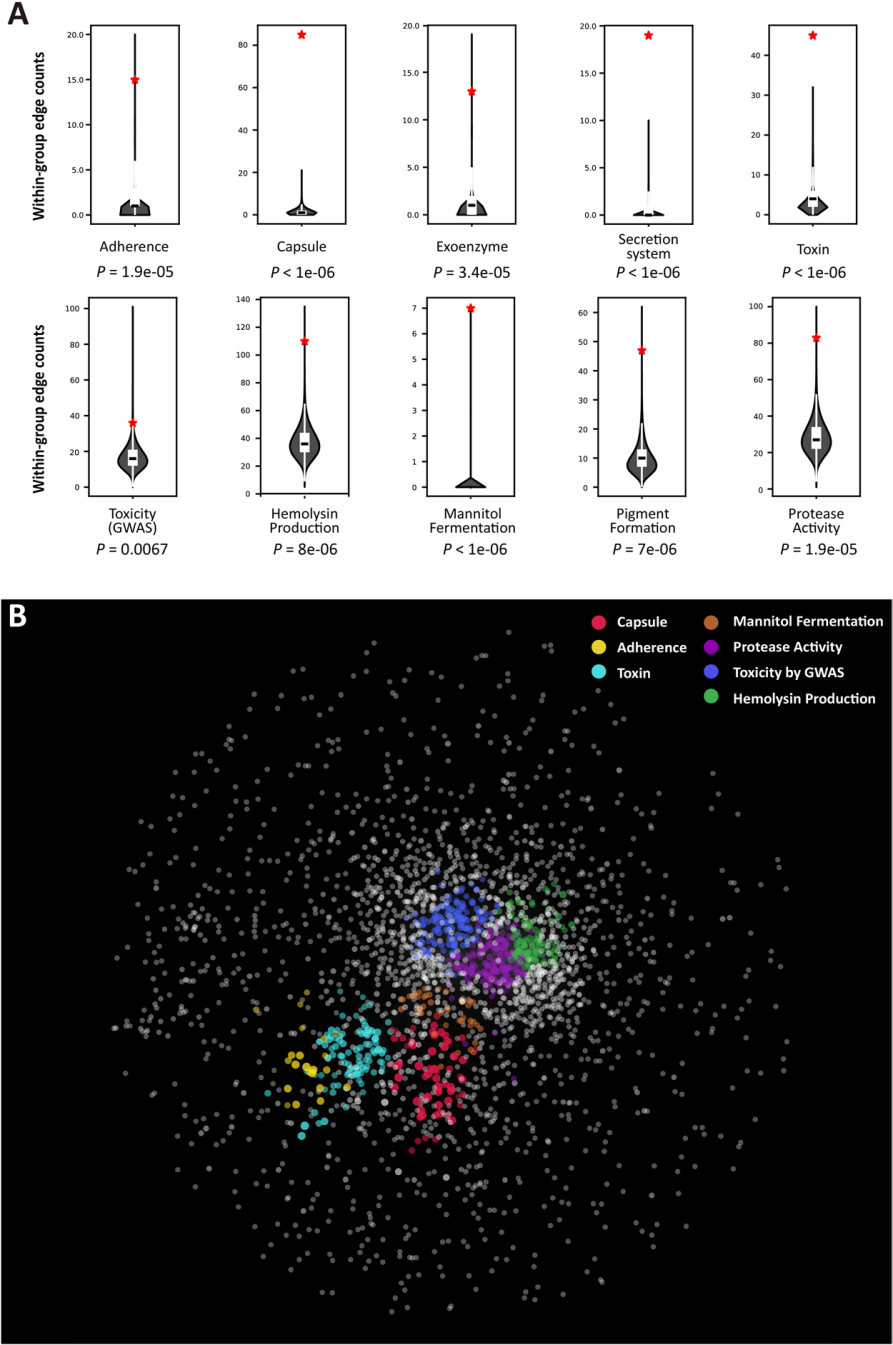
Genes for virulence-associated phenotypes are interconnected in StaphNet. (**A**) For various virulence-associated phenotypes, the within-group edge count in StaphNet, which means the number of the edges between genes for the same phenotype, was compared with that of the same size of random gene sets. For each phenotype, a violin plot and an inner box plot represent the distribution of the within-group edge counts by a million random gene sets. Red stars represent the within-group edge counts for the given virulence-associated phenotypes. (**B**) The SAFE algorithm was used to detect the local enrichment modules for seven virulence-associated phenotypes in StaphNet. The various phenotypes are represented by different colors.

To form a predictive network, the genes for a phenotype need to be more closely connected to each other in the network than they would be by random chance. To determine whether the genes for the same virulence phenotypes were significantly interconnected in StaphNet, we compared the number of direct connections among the genes for the same virulence phenotype with the number of direct connections among the same number of random genes. For all the virulence-associated phenotypes in our analysis, we found that the genes for each phenotype were highly interconnected in StaphNet (*P*-value < 1 × 10^−4^ for all tested phenotypes except GWAS toxicity, which had a *P*-value = 0.0067, according to a permutation test of 10^6^ randomized samples) (Fig. 2A). We also visualized the modularity of virulence phenotype genes in StaphNet using SAFE software, which finds and visualizes local enrichment for function in a given network^30^. We found that 7 out of 11 virulence phenotypes—capsule, adherence, toxin, mannitol fermentation, protease activity, GWAS toxicity, and hemolysis production—showed significant region-specific enrichment (enrichment *P*-value < 0.05) in StaphNet (Fig. 2B).

### StaphNet implementations for network-based identification of novel virulence genes

**Figure 3:**
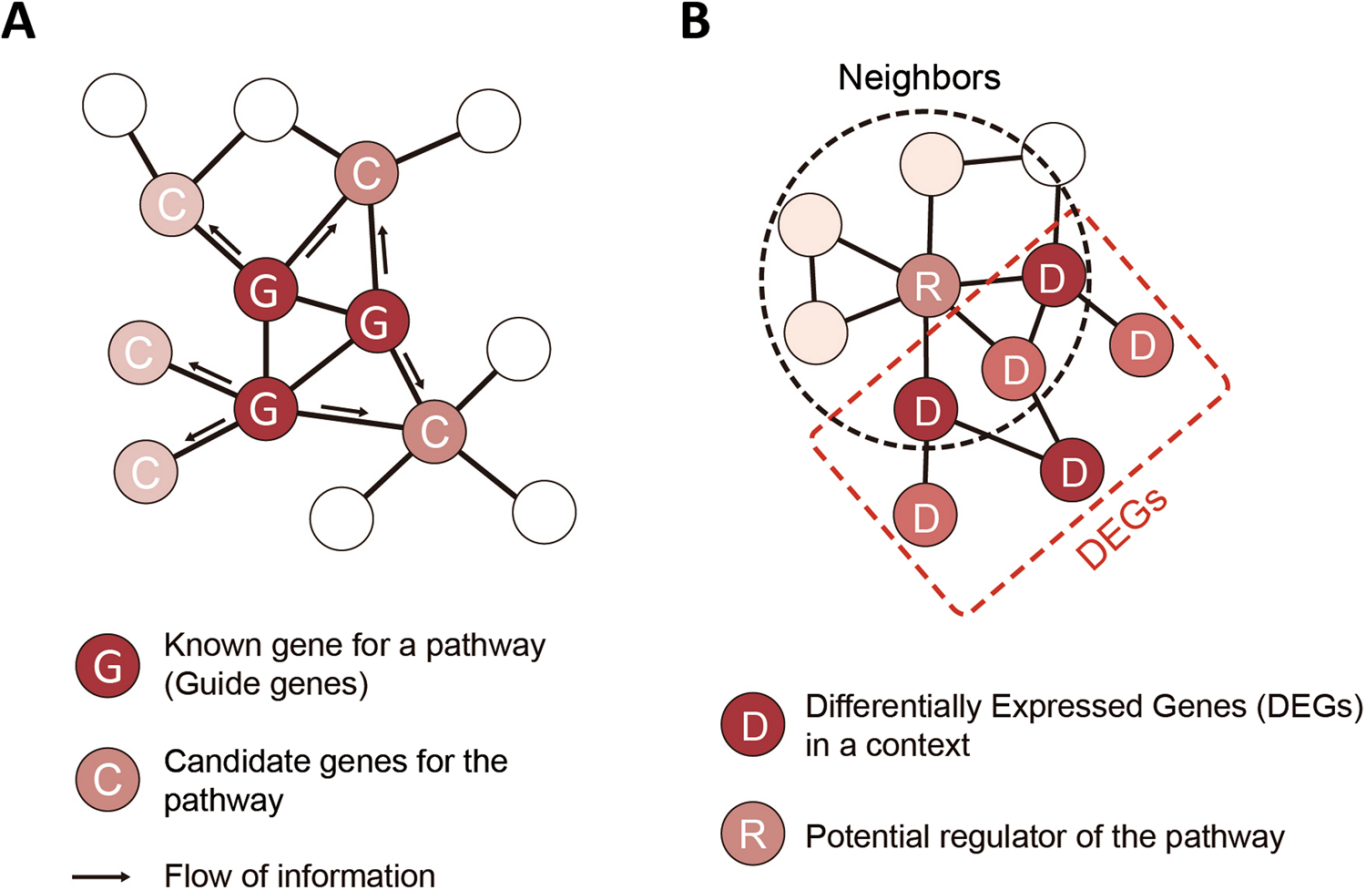
Schematic diagrams of network-based gene prioritization algorithms implemented in StaphNet server. (**A**) Pathway-centric search algorithm. (**B**) Context-centric search algorithm.

To increase the utility of StaphNet, we implemented two network-based gene prioritization algorithms in a companion web application (www.inetbio.org/staphnet): pathway-centric search and context-centric search. The pathway-centric search algorithm is an efficient tool for searching new candidate genes for a pathway or phenotype of interest. Users submit “guide genes”, which are known genes for a pathway or phenotype of interest, to the StaphNet network search page. StaphNet then propagates pathway or phenotype labels from the guide genes to the neighboring genes. The neighboring genes are then ranked according to the highest label score propagated from the guide genes (Fig. 3A). Neighbor genes that connect to more guide genes with stronger edge weights rank higher. StaphNet reports top 100 candidate genes based on sum of log likelihood scores (see the Methods) of edges that connect to all guide genes. It also reports contribution of each type of co-functional associations that support the total prediction score. The context-centric search algorithm initiates a network search of differentially expressed genes (DEGs) for a biological context of interest. When users submit DEGs for a certain context relevant to a pathway or phenotype of interest, the algorithm looks for hub genes (degree ≥ 20) whose neighbors are significantly overlapped with the DEGs (Fig. 3B). Thus, the context-centric search algorithm suggests hub genes that are likely to be associated with the context.

### StaphNet identified a novel gene for hemolysis activity

**Figure 4:**
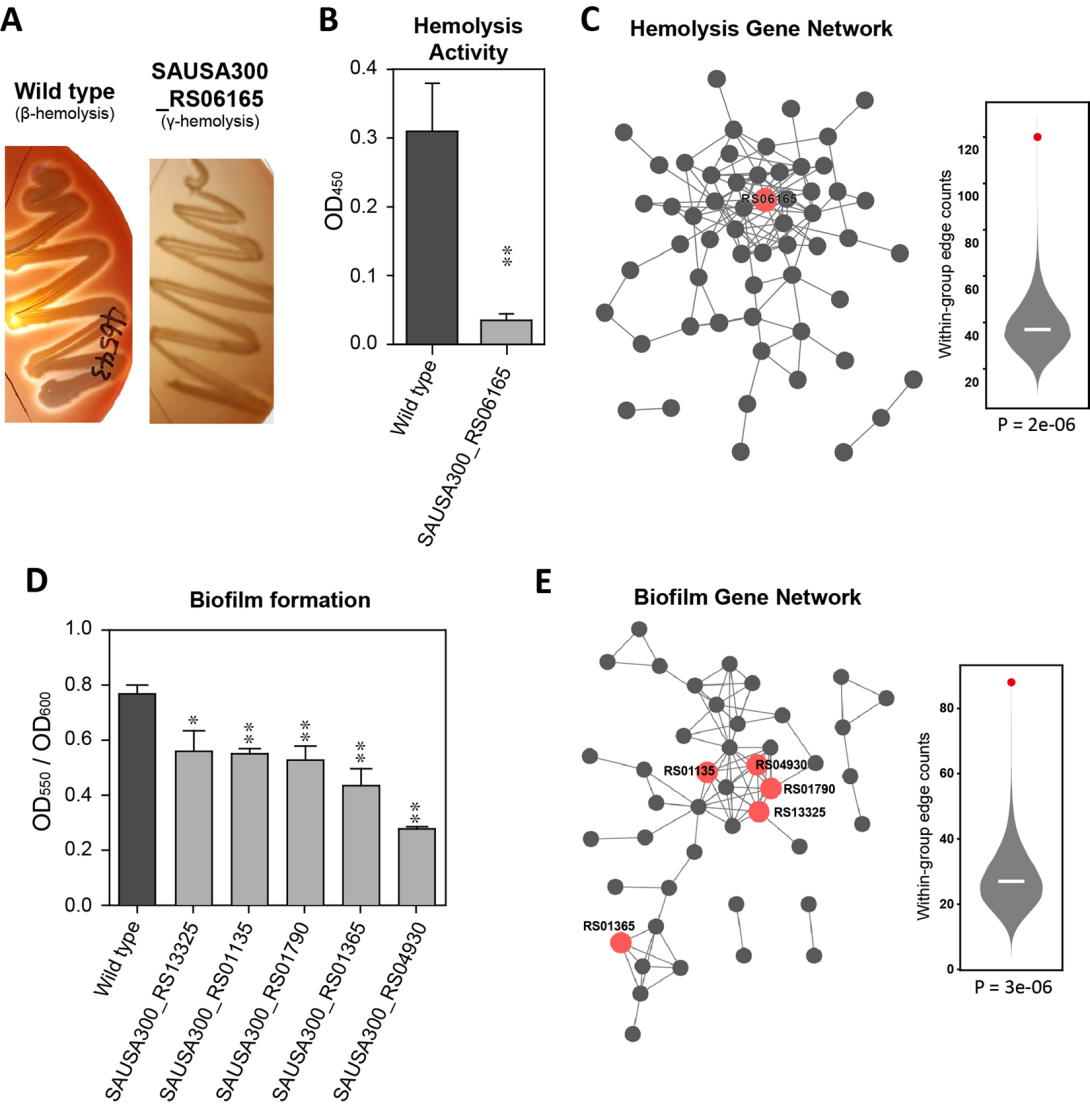
Network-based identification of novel genes for virulence-associated phenotypes. (**A**) The blood agar plate streaked with wild-type *Staphylococcus aureus* exhibited beta-type hemolysis, whereas the plate streaked with the mutant *S*. *aureus* with the SAUSA300_RS06165 gene exhibited gamma-type hemolysis. (**B**) Hemolysis activity (OD_450_) of the wild-type *S*. *aureus* and the mutant *S*. *aureus* with the SAUSA300_RS06165 gene. Error bars represent the standard error, and the double asterisk signifies a *P*-value ≤ 0.01 (two-tailed t-test). (**C**) Hemolysis gene network including a novel hemolysis gene, RS06165 (red node) and its within-group connectivity score (red dot). Significance of the observed network connectivity was measured by using a null distribution based on 10000 random gene sets. (**D**) Biofilm-forming capability was normalized to the bacterial growth (OD_550_/OD_600_) of the wild-type and mutant *S*. *aureus*. Error bars represent the standard error, the single asterisk represents a *P*-value ≤ 0.05, and the double asterisks represent *P*-values ≤ 0.01 (two-tailed t-test). (**E**) Biofilm gene network including five novel biofilm genes (red nodes) identified in this study and its within-group connectivity score (red dot). Significance of the observed connectivity was tested as for (**C**).

We tested the feasibility of network-based identification of virulence-associated phenotype genes using the pathway-centric search algorithm of StaphNet. *S*. *aureus* produces alpha-hemolysins, which form pores in the membranes of the host erythrocytes. These pores induce unwanted ion exchange, which leads to cell death. Therefore, the hemolysis activity of bacteria is a promising target for attenuating virulence. There have been reports that knockout of the alpha-hemolysin gene (*hla*) leads to a reduction in the invasiveness and virulence of *S*. *aureus*^31^. Searching for novel genes for hemolysis activity, we performed a pathway-centric search on the StaphNet web server using 71 previously reported hemolysis genes^28^ as guide genes. We prioritized *S*. *aureus* genes for hemolysis activity based on the sum of network edge scores (log likelihood score as described in the Methods) to all connected guide genes. Among the predicted candidate genes, we selected the top 10 genes with available mutant strains from the Biodefense and Emerging Infections Research Resources Repository (BEI Resources) (https://www.beiresources.org/) (Table 3). We then investigated the hemolysis type of the mutant strains by streaking the bacteria on blood agar plates (see the Methods section for more details). Among the 10 tested genes, SAUSA300_RS06165, which encodes succinyl-CoA ligase subunit alpha, produced the gamma-hemolysis (non-hemolytic) phenotype in a mutant strain, whereas the wild-type strain exhibited the beta-hemolysis (complete hemolysis) phenotype (Fig. 4A). We also quantified hemolysis arising from SAUSA300_RS06165 (see the Methods section for more details) and found a significant reduction in hemolysis activity in the mutant strain (*P*-value = 0.0013, two-tailed t-test) (Fig. 4B). As expected, the novel hemolysis gene SAUSA300_RS06165 was highly connected to the guide genes, and the updated network of hemolysis genes showed significantly higher within-group edge count score than those for random gene sets (Fig. 4C). These results demonstrate that the pathway-centric search algorithm can identify novel genes for virulence-associated phenotypes in *S*. *aureus*.

**Table 3:** Candidate gene list for hemolysis activity by pathway-centric search.

Rank	Gene ID (New locus tag)	Pathway-Centric Search Score	Mutant Strain Availability (Tested Rank)	Significant Change in Hemolysis Ability
1	SAUSA300_RS11335	24.58	TRUE (1)	FALSE
2	SAUSA300_RS05175	22.24	TRUE (2)	FALSE
3	SAUSA300_RS07105	22.04	TRUE (3)	FALSE
4	SAUSA300_RS11410	20.84	FALSE	Not tested
5	SAUSA300_RS11425	20.64	TRUE (4)	FALSE
6	SAUSA300_RS07100	19.34	TRUE (5)	FALSE
7	SAUSA300_RS11990	18.85	FALSE	Not tested
8	SAUSA300_RS01250	18.38	TRUE (6)	FALSE
9	SAUSA300_RS11355	18.36	TRUE (7)	FALSE
10	SAUSA300_RS02850	18.23	FALSE	Not tested
11	SAUSA300_RS07405	17.96	TRUE (8)	FALSE
12	SAUSA300_RS07430	17.58	FALSE	Not tested
13	SAUSA300_RS12145	17.27	FALSE	Not tested
14	SAUSA300_RS11400	17.06	FALSE	Not tested
15	SAUSA300_RS11380	16.85	TRUE (9)	FALSE
16	SAUSA300_RS02805	16.66	FALSE	Not tested
17	SAUSA300_RS09090	16.66	FALSE	Not tested
18	SAUSA300_RS06220	16.59	FALSE	Not tested
**19**	**SAUSA300_RS06165**	**16.02**	**TRUE (10)**	**Gamma type hemolysis, Quantitative assay p < 0.01**

### StaphNet identified novel genes for biofilm formation

Next, we investigated context-centric searching for the identification of virulence-associated phenotype genes. When they form biofilms, bacterial cells are able to settle close to each other and cooperate in harsh host microenvironments^32^. Bacterial cells that reside in the inner part of a biofilm are physically separated from anti-microbial agents, and can, therefore, survive antibiotic treatments^33^. To predict novel candidate genes for biofilm formation in *S*. *aureus*, we compiled a list of 56 genes with expression levels that increase by more than twofold in biofilms compared with in stationary-phase planktonic culture^34^, and submitted them as input data for a context-centric search of the web server. Then we prioritized *S*. *aureus* genes for biofilm formation based on significance of overlap between their neighboring genes in StaphNet and the 56 genes that were up-regulated in biofilms. We selected the top 10 predicted candidate genes with available mutant strains (Table 4). We then determined the biofilm-forming ability of the mutant and wild-type strains. Because the number of bacterial cells dictates the quantity of biofilm, we normalized the amount of biofilm to the number of bacterial cells (see the Methods section for more details). Notably, 5 of the 10 tested candidate genes apparently had mutant strains with reduced biofilm-forming ability (*P*-value < 0.05 for SAUSA300_RS13325, and *P*-values < 0.01 for SAUSA300_RS01135, SAUSA300_RS01790, SAUSA300_RS01365, and SAUSA300_RS04930, according to a two-tailed t-test) (Fig. 4D). The five novel genes for biofilm formation are connected to many up-regulated genes in biofilms and their network has significantly higher within-group edge count score than those for random gene sets (Fig. 4E). These results indicate that the context-centric search algorithm is highly effective for the identification of phenotypic genes in *S*. *aureus*.

**Table 4:** Candidate gene list for biofilm forming ability by context-centric search.

Rank	Gene ID (New locus tag)	Context-Centric Search p-value	Mutant Strain Availability (Tested Rank)	Significant Change in Biofilm Forming Ability (Significance)
1	SAUSA300_RS12890	1.73569E-08	FALSE	Not tested
2	SAUSA300_RS12950	9.84818E-08	TRUE (1)	FALSE
3	SAUSA300_RS12940	1.04527E-05	TRUE (2)	FALSE
4	SAUSA300_RS12935	3.05251E-05	FALSE	Not tested
5	SAUSA300_RS03540	3.89378E-05	FALSE	Not tested
6	SAUSA300_RS04955	5.47043E-05	FALSE	Not tested
7	SAUSA300_RS12930	0.000143066	FALSE	Not tested
**8**	**SAUSA300_RS13325**	**0.00019952**	**TRUE (3)**	**TRUE (p < 0.05)**
9	SAUSA300_RS12695	0.000271935	FALSE	Not tested
10	SAUSA300_RS09310	0.000287212	TRUE (4)	FALSE
11	SAUSA300_RS12380	0.000384348	FALSE	Not tested
12	SAUSA300_RS07240	0.000402737	TRUE (5)	FALSE
13	SAUSA300_RS00945	0.000498033	FALSE	Not tested
**14**	**SAUSA300_RS01135**	**0.000510171**	**TRUE (6)**	**TRUE (p < 0.01)**
15	SAUSA300_RS04750	0.000510171	FALSE	Not tested
**16**	**SAUSA300_RS01790**	**0.000673005**	**TRUE (7)**	**TRUE (p < 0.01)**
**17**	**SAUSA300_RS04930**	**0.000754106**	**TRUE (8)**	**TRUE (p < 0.01)**
18	SAUSA300_RS14020	0.0014589	FALSE	Not tested
19	SAUSA300_RS11780	0.001491269	FALSE	Not tested
20	SAUSA300_RS06610	0.001493588	TRUE (9)	FALSE
21	SAUSA300_RS13915	0.001493588	FALSE	Not tested
22	SAUSA300_RS00475	0.001826731	FALSE	Not tested
23	SAUSA300_RS00625	0.002214592	FALSE	Not tested
24	SAUSA300_RS13275	0.002659488	FALSE	Not tested
25	SAUSA300_RS01860	0.003166104	FALSE	Not tested
**26**	**SAUSA300_RS01365**	**0.003214458**	**TRUE (10)**	**TRUE (p < 0.01)**

### Availability of StaphNet data and web applications

All edge information for StaphNet, component networks, and gold-standard gene pairs is freely available at www.inetbio.org/staphnet. Two network prediction algorithms—pathway-centric search and context-centric search—can be performed by the submission of guide genes and DEGs, respectively. Although StaphNet performs network searches based on a gene network of *S*. *aureus* subsp. aureus USA300_FPR3757, the network search is also compatible with another 30 *S*. *aureus* strains. For example, if users submit NCTC8325 genes known for virulence-associated phenotypes, the web server uses orthologous USA300 strain genes for the network search. All candidate genes are reported by both USA300 genes and NCTC8325 genes. For orthologous gene mapping of major MRSA strains such as N315, MRSA252, NCTC8325, and TW20, we used bidirectional best hits according to BLASTP (BLASTP stands for the Basic Local Alignment Search Tool for Proteins). For all other strains, we adopted the orthologous relationships between locus tags of different strains from AureoWiki (http://aureowiki.med.uni-greifswald.de/). AureoWiki was used to assign orthologous relationships between genes having more than 50% DNA sequence identity and more than 70% protein identity.

## Discussion

In this study, we presented a network-based framework for genetic investigation of phenotypes in the gram-positive bacterial pathogen MRSA. Although we focused on virulence-associated phenotypes, the same method can be applied to other phenotypes. Effective network-based prediction requires two components: an accurate network and a network analysis algorithm. StaphNet, which is an integrated co-functional network, exhibited high accuracy and predictive power for virulence phenotypes in computational assessments. Experimental validation provided further support for the usefulness of StaphNet in genetic investigations of virulence-associated phenotypes in MRSA. We tested two alternative network-based functional prediction algorithms in this study. The pathway-centric search algorithm relies on network connectivity among genes for the same pathway or phenotype. StaphNet had high predictive power for known genes for various virulence-associated phenotypes. However, we were only able to experimentally validate 1 out of 10 tested candidate genes for hemolysis activity. This can be attributed to the incomplete penetrance of mutations of the novel candidate genes for hemolysis, so the phenotypic effect was undetectable using a simple blood agar plate assay. It is possible that most of the core genes for hemolysis have been identified already, and only genes that are weakly associated with the phenotype remain undiscovered. We may conduct blood agar plate assays with double mutations for the candidate genes to test this hypothesis. In contrast, we observed a high proportion of true positives for the prediction of biofilm formation using the context-centric search algorithm. This may have arisen because all the candidates for this algorithm were network hubs (degree ≥20), which are more likely to be functionally associated with many other genes. Given that hub genes tend to be essential, predictions with hub genes are more likely to exhibit the phenotypic effects of mutation. This suggests that the context-centric search algorithm could be more effective than the pathway-centric search algorithm in searching for genes with phenotypic effects.

We observed that StaphNet was very efficient at predicting GWAS candidate genes that increase toxicity. Historically, the major forward-genetics method used for microbial species was random mutagenesis library screening. However, as more strains with completely sequenced genomes become available, another forward-genetics method, GWAS, is being used for several major human infectious microbes, including MRSA^35^. In human GWASs, the biological relevance of GWAS candidates has been tested by assessing the significance of network interactions between candidate genes^36^. We observed significant interconnectivity in candidate genes from GWAS that increase toxicity in StaphNet, which indicates that it may also be possible to use gene networks to test the biological relevance of GWAS candidates in microbes. Moreover, GWAS candidates can be prioritized^37^ or augmented^38^ by functional gene networks. The development of similar network applications for microbial GWAS candidates may be warranted in the future.

## Methods

### Construction of the StaphNet genome and gold-standard functional gene pairs of *S*. *aureus subsp*. *aureus USA300_FPR3757*

We downloaded the genome sequence of MRSA *subsp*. *aureus USA300_FPR3757* from the National Center for Biotechnology Information (NCBI) genome database as of February 19, 2016. We excluded pseudogenes from the list of downloaded genes. The final gene set used for the network construction contained 2,845 protein-coding genes. Coding genes for other major MRSA strains such as N315, MRSA252, NCTC8325, and TW20 were also downloaded from the NCBI genome database at the same time.

We constructed the StaphNet using a supervised machine learning approach. To train the network model, we compiled gold-standard co-functional gene pairs from molecular pathways annotated by KEGG^22^ (as of February, 2016) and MetaCyc^23^ (as of February 2016) databases by pairing genes that were annotated using the same pathway terms. For the KEGG database, we ignored “global pathways”, which may harbor multiple sub-pathways, resulting in many between-pathway gene pairs. The relatively large size of the global pathways may also result in biased gold-standard gene pairs owing to the exponential increase in the number of gene pairs as the number of genes increases. Using these pathway filtrations, we were able to generate 20,258 gold-standard gene pairs from 930 genes.

### Benchmarking and integrating co-functional networks

We benchmarked and scored networks from distinct genomics data sets and integrated them using a log likelihood score (*LLS*) scheme based on Bayesian statistics^24^. We calculated the *LLS* score using the following equation:

$$LLS=\,\mathrm{ln}(\frac{P(L|E)/P(\neg L|E)}{P(L)/P(\neg L)})$$

where *P*(*L*|*E*) represents the probability of positive gold-standard links for the given genomic evidence (E), and $P(\neg L|E)$ represents the probability of negative gold-standard links for the same genomic evidence. *P*(*L*) and $P(\neg L)$ represent the probability of positive and negative gold-standard links, respectively.

Different genomics data may exhibit some correlation. Therefore, naïve Bayesian integration of multiple *LLS*s for each co-functional link would be suboptimal. We previously devised a weighted sum (*WS*) method for data integration, which can handle data correlation to some extent by applying different weights during summing-up of the multiple edge scores using the following equation:

$$WS={S}_{0}+\,\sum _{i=1}^{n}\,\frac{{S}_{i}}{D\times i},\,for\,all\,S\ge T$$

where *S*_0_ represents the highest *LLS*, *S*_*i*_ represents the *i*th highest *LLS* after the second highest *LLS*, and *D* is a free parameter that adds a decrementing weight to each score. *T* indicates the threshold of the minimum *LLS* to be integrated. We chose the free parameters where the integrated network achieved the highest benchmarking score. All networks inferred from individual genomics data sets and from component networks of distinct data types (Table 2) were integrated using the benchmarking and integration pipeline described above. The final integrated network, StaphNet, contains 60,513 links and 2,674 genes (94% of the coding genes) in MRSA strain USA300.

### Co-functional networks inferred by co-expression analysis of *S*. *aureus* genes (SA-CX)

Genes that exhibit similar expression profiles across various conditions tend to be co-regulated for the same pathway. We measured the co-expression between two genes using the Pearson correlation coefficient. We obtained gene expression data from the Gene Expression Omnibus (GEO)^39^ database. We performed co-expression analysis for each GEO series (GSE) and generated a total of 24 co-expression networks, of which 20 GSEs were based on microarray platforms and four GSEs on RNA sequencing (Table 1). For the sequencing-based expression data, we performed a preprocess on short reads using an alignment-free quantification algorithm, kallisto^40^ (version 0.42.4), which gives transcripts per million (TPM) values. We benchmarked and integrated the 24 co-expression networks into a single co-functional network by co-expression analysis.

### Co-functional networks inferred by similarity of domain profiles (SA-DP)

Protein domains are the structural and functional units of proteins. Therefore, two protein-coding genes that share similar domains (i.e., similar domain profiles) are likely to have similar functions. We constructed the domain profiles of *S*. *aureus* genes using domain information from the InterPro database^41^. We measured the similarity between domain profiles using weighted mutual information (WMI) scoring^20^, which takes account of the different information weights among domains during the mutual information (MI) calculation. We assigned greater weights to rarer domains, assuming there is more specific information on molecular pathways for rarer domains. After benchmarking, we obtained a co-functional network according to domain profile similarity.

### Co-functional networks inferred by similarity of phylogenetic profiles (SA-PG)

Organisms gain and lose genes during speciation. Genes for the same functional pathways tend to be co-inherited during speciation. Therefore, similar phylogenetic profiles between two genes across diverse species implies their functional association. To measure phylogenetic profile similarity among *S*. *aureus* genes, we constructed phylogenetic profiles for each *S*. *aureus* gene based on the BLASTP hit score to reference genomes for other species. The profile similarity was measured by MI. We previously found that phylogenetic profiles for each of the three domains of life (Archaea, Bacteria, Eukarya) enabled the detection of co-inheritance patterns within each domain^19^. The three networks inferred from the domain-specific profiles are complementary, allowing network improvement by integration. We therefore inferred networks from three individual phylogenetic profiles based on 1,626 bacteria, 122 archaea, and 396 eukaryotes. After benchmarking and integration of the three inferred networks, we obtained a single co-functional network according to phylogenetic profile similarity.

### Co-functional networks inferred by gene neighborhood (SA-GN)

In the prokaryotic genome, functionally associated genes often exist in proximity, forming a co-transcriptional unit known as an operon. Therefore, we may infer functional coupling between genes if their orthologs reside in the same neighborhood in prokaryotic genomes. We used two complementary methods to infer gene neighborhood in 1,749 prokaryotic genomes: probability-based gene neighborhood (PGN) and distance-based gene neighborhood (DGN)^18^.

We extended the gene neighborhood approach to metagenome sequence data^42^. A massive amount of sequence data has recently become available as a result of metagenomic shotgun sequencing technology. The majority of the identified gene models in the database are not annotated bacterial genes, because there are many bacterial species that cannot be cultured under laboratory conditions. Therefore, metagenomes with novel gene models are a potential new resource for gene neighborhood analysis. We downloaded the metagenome contigs for the 16 human body sites from the Human Microbiome Project^43^, and global samples from the TARA Oceans project^44^. We aligned the *S*. *aureus* genes to the metagenome assemblies using the ultra-fast sequence alignment algorithm, DIAMOND^45^. Because PGN requires completely assembled genomes, we applied DGN for metagenome data only. The gene neighborhood score for each gene pair (*S*) was calculated using the following equation:

$$S=\,\mathrm{log}(\frac{d+1}{n})$$

where *n* is the total number of contigs in which two genes co-occur and *d* is the median distance (bp) between them across all co-occurring contigs. Finally, three networks, the PGN, the DGN, and the metagenome-based gene neighborhood (MGN), were benchmarked and integrated into a single co-functional network according to gene neighborhood.

### Co-functional networks based on high-throughput protein–protein interaction (PPI) assays (SA-HT)

We compiled 13,219 PPIs among 608 MRSA proteins based on a high-throughput protein complex pull-down assay^17^. We generated network links between prey and baits for each pull-down complex (i.e., the spoke-model). Using a binary score scheme (1 for interaction and 0 for no interaction), we generated PPI profiles for each MRSA gene. We then determined MI between genes using the PPI profiles. With the benchmarking method, we empirically determined that this link-scoring scheme gave the best co-functional network. We ultimately found 6,480 PPIs among 355 genes that were likely to be functionally coupled.

### Co-functional networks transferred from *Escherichia coli* (EC-CX and EC-HT)

We were able to infer co-functional links between *S*. *aureus* genes by orthology-based gene pair transfer from *E*. *coli*, which has a well-established co-functional network, EcoliNet^21^. We transferred individual component networks from EcoliNet by orthologous gene pairs (associalogs^46^) between *S*. *aureus* genes and *E*. *coli* genes detected using Inparanoid software^47^. We assigned Inparanoid-weighted LLSs (IWLLSs) to each associalog using the following equation:

$$IWLLS({\rm{A}}-{\rm{B}})=\,\mathrm{ln}\,[Inparanoid\,score\,({\rm{A}},{\rm{A}}^{\prime} )]+\,\mathrm{ln}\,[Inparanoid\,score\,({\rm{B}},{\rm{B}}^{\prime} )]+LLS({\rm{A}}^{\prime} -{\rm{B}}^{\prime} )$$

where A and B are *S*. *aureus* genes and A′ and B′ are their orthologs in *E*. *coli*.

With benchmarking analysis, we found that associalogs from the *E*. *coli* co-expression network and the *E*. *coli* high-throughput PPI network produced co-functional networks of MRSA genes.

### Analysis of network centrality

Degree centrality for the gene *i* represents the number of direct neighbors of gene *i*. For a given gene *i*, betweenness centrality represents the number of shortest paths among the genes in the network that pass through the gene *i*. We ignored the edge weight for the betweenness centrality calculation. We used the igraph R package (http://igraph.org/) for the centrality calculation.

### Network visualization

We employed SAFE^30^ for network visualization to find local enrichment patterns of virulence genes in the global network. We visualized the entire StaphNet using Cytoscape^48^ with “edge-weighted spring embedded” layout, neighborhoodRadiusType and groupDistanceThreshold options, and parameters of 7.5 and 0.5 for diameter and neighborhoodRadius, respectively.

### Hemolysis assay

We determined the hemolysis types of 11 strains (10 candidate mutant strains and a wild-type control) by streaking the bacterial cells on sheep blood agar plates. We selected a strain with a positive result from the blood agar plate assay for further quantitative investigation of hemolysis. We measured the hemolysis capability of each strain using the method previously described^49,50^. Briefly, the bacterial cells were cultured overnight, then sub-cultured in 10 mL of tryptic soy broth (TSB) for 3 h at 37 °C (optical density at 600 nm (OD_600_) ≈ 0.6). After centrifugation of the bacterial culture at 13,000 rpm for 1 min at 4 °C, a 100-μL aliquot of the supernatant was mixed with 900 μL of 8% sheep blood, which was subsequently washed three times in phosphate-buffered saline (PBS). The resultant mixtures were incubated at 37 °C for 3 h, then centrifuged at 1,500 *g* for 10 min at 4 °C. We then measured the OD_450_ for each strain using a BioTek Epoch spectrometer.

### Biofilm formation assay

We measured the biofilm formation capability of each strain using the method previously described^51^. In brief, the strains were cultured overnight and diluted 1:100 with the TSB medium. Aliquots of the diluted culture (100 μL) were dispensed onto 96-well plates. We prepared two identical 96-well plates to normalize the growth rate of the bacteria. For one plate, we measured the OD_600_, and for the other plate, we removed the unattached bacteria by dumping and washing. We added 125 μL of 0.1% crystal violet (CV) solution and incubated at room temperature for 15 min. After dumping out the staining solution, we dried the plate for 24 h. We added 125 μL of acetic acid to the plate to solubilize the CV and incubated at room temperature for 15 min. We then measured the OD_550_ of each strain using a BioTek Epoch spectrometer. For each strain, we divided the OD_550_ by the OD_600_ to normalize the growth rate of each strain. We chose five strains to generate replicates.
